# BRAF mutation and its inhibitors in sarcoma treatment

**DOI:** 10.1002/cam4.3103

**Published:** 2020-05-31

**Authors:** Haotian Liu, Nahar Nazmun, Shafat Hassan, Xinyue Liu, Jilong Yang

**Affiliations:** ^1^ Department of Bone and Soft Tissue Tumor Tianjin Medical University Cancer Institute & Hospital Tianjin P.R. China; ^2^ National Clinical Research Center for Cancer Tianjin Medical University Cancer Institute & Hospital Tianjin P.R. China; ^3^ International Medical School Tianjin Medical University Tianjin P.R. China

**Keywords:** BRAF inhibitors, BRAF mutation, Sarcoma, targeted therapy

## Abstract

The mitogen‐activated protein kinase (*MAPK*) signaling pathway plays a significant role in mediating cellular physiological activities, such as proliferation, differentiation, apoptosis, and senescence. This signaling pathway is composed of several major proto‐oncogenes of *RAS/RAF/MEK/ERK*, among which the *BRAF* proto‐oncogene, as one of the three members of the *RAF* family, has a higher mutation rate than *ARAF* and *CRAF* and has attracted extensive attention. Regarding the BRAF mutation, approximately 95% of BRAF mutations belong to the BRAF V600E mutation, which can enhance the expression of the *MAPK* signaling pathway and is thus related to the occurrence and development of various malignant tumors and has been successfully identified as a therapeutic target. Moreover, drug resistance to BRAF inhibitor treatment also appears to be an important issue. Considering the successful use of BRAF inhibitors in melanoma, we provide a brief overview of the BRAF mutations, including their basic structures and activation mechanisms, and the new classification method for BRAF mutations. Most importantly, we summarize the results of BRAF inhibitor treatment in different sarcomas. To overcome drug resistance to BRAF inhibitor treatment, we also outline the different mechanisms of drug resistance to BRAF inhibitor treatment and introduce the combination strategy of BRAF inhibitors with other targeted therapies.

## Graphical abstract

BRAF structures and their activation mechanisms.

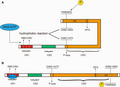

## 1 INTRODUCTION

**Figure 1:**
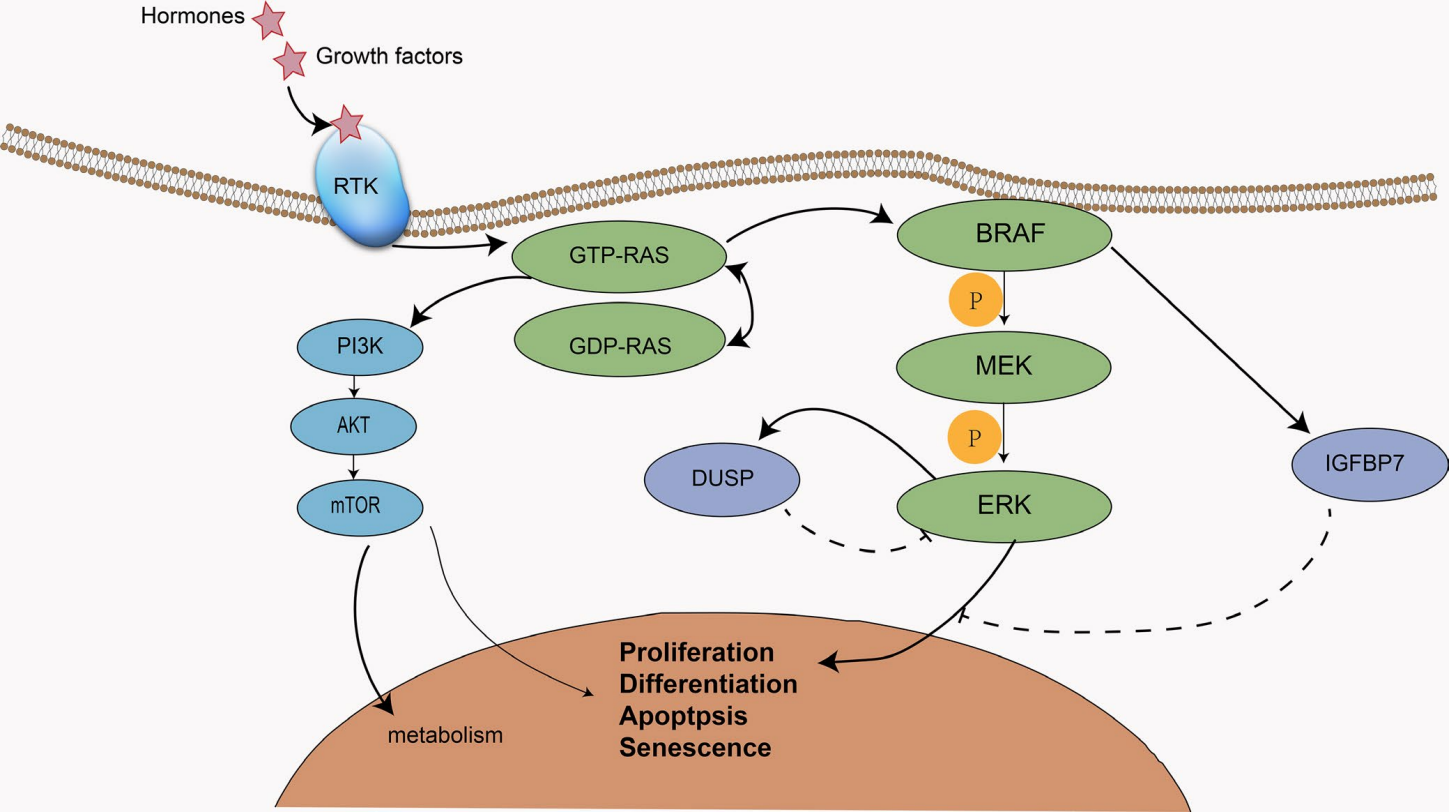
The *MAPK* and *PI3K‐AKT‐mTOR* signaling pathways. In addition to activating the *MAPK* signaling pathway, *RAS* also activates the *PI3K‐AKT‐mTOR* signaling pathway. The *PI3K* signaling pathway can also promote metabolism, while the *MAPK* pathway is more active in cell proliferation. There are several negative feedback regulatory mechanisms in the BRAF mutation *MAPK* signaling pathway. For example, BRAF secretes IGFBP7, which can suppress the ERK signaling pathway paradoxically and lead to cell senescence and apoptosis. In addition, ERK activates DUSP, which dephosphorylates ERK and inhibits ERK activity

The mitogen‐activated protein kinase (*MAPK*) pathway, consisting of *RAS/RAF/MEK/ERK* can transfer extracellular signals, including hormones, cytokines, and growth factors, to the nucleus, thus changing gene expression in the cell and mediating proliferation, differentiation, survival, and apoptosis.^1^, ^2^, ^3^, ^4^, ^5^ *RAS* comprises three isoforms: *KRAS*, *NRAS*, and *HRAS*.^4^, ^6^ It can be transformed between the state of active GTP‐bound and the state of inactive GDP‐bound.^7^ RAS activates at least 10 downstream signaling pathways, of which the classic one is *RAS/RAF/MEK/ERK*.^6^, ^7^ Extracellular signaling molecules activate RAS by binding to a receptor tyrosine kinase (RTK), and active RAS recruits RAF to the plasma membrane. When RAF is activated on the cell membrane, it phosphorylates downstream MEK, which phosphorylates ERK, thereby producing biological effects^1^, ^3^, ^4^ (Figure 1).

Abnormal activation of the *MAPK* pathway is partly caused by mutations in RAS and RAF, which changes the normal physiological activities of cells, promoting growth and differentiation and is related to the development of a variety of tumors. For example, RAS mutation is associated with pancreatic cancer, lung cancer, and colorectal cancer, while RAF mutation can be detected in melanoma, thyroid cancer, and other malignant tumors.^6^ Concerning the RAF family, the BRAF mutation has attracted extensive attention due to its extensive mutation phenomenon in a variety of tumors and its higher mutation rate compared with ARAF and CRAF. In this article, we review the therapeutic efficacy of BRAF inhibitors in sarcomas, and summarize the mechanism of resistance to BRAF inhibitors and the combination with other targeted drugs.

## 2 THE BRAF ONCOGENE

**Figure 2:**
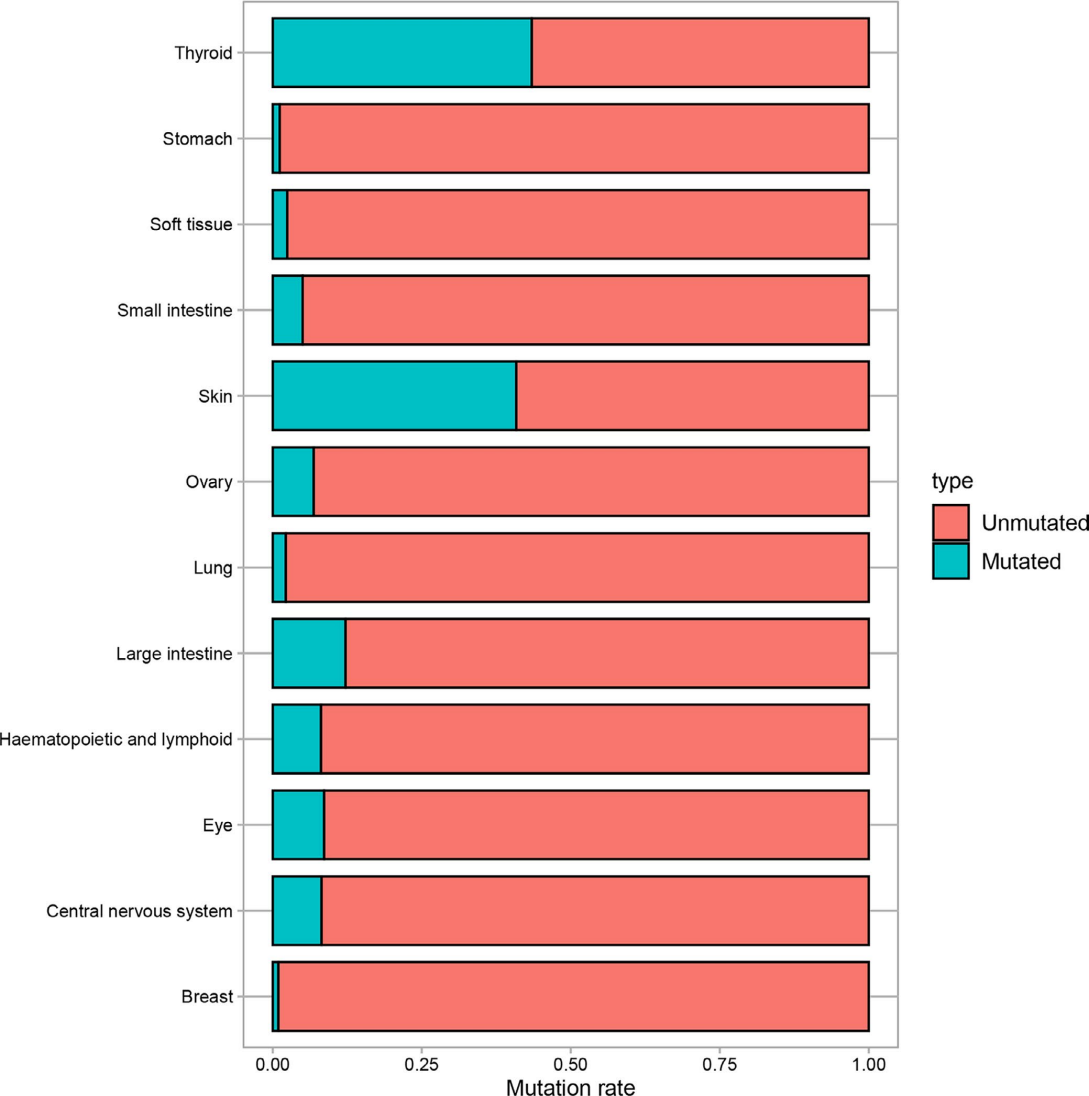
Distribution of BRAF mutations in different tissues

*RAF* comprises three isoforms: *ARAF*, *BRAF*, and *CRAF* (also known as *V‐RAF*).^1^ Understanding of BRAF was achieved mainly through the study of CRAF because they have substantial sequence homology.^2^ In fact, in the *MAPK* pathway signaling process, there is a special relationship between BRAF and CRAF, that is, BRAF can not only directly activate MEK but also activate MEK by activating CRAF; however, in turn, CRAF cannot activate BRAF.^8^ This may explain the mechanism of BRAF inhibitor resistance to some extent. Since the BRAF mutation was identified in 2002, more than 50 mutations have been reported, and different tissues have different mutation frequencies^9^, ^10^(Figure 2). Ninety‐five percent of these mutations result from a kind of missense mutation in exon 15, that is, thymine mutates into adenosine at nucleotide 1799 (T > A), which contributes to changes in protein expression levels—valine (V) replaces glutamic acid (E) at amino acid 600.^1^, ^4^, ^5^ For this reason, this mutation is called BRAF V600E.^11^ BRAF V600E increases the activity of BRAF by 500 times, leading to an increase in MEK and ERK activity.^1^ Furthermore, this mutant does not need to bind to RAS to activate ERK, that is, it is a pattern of activation that does not depend on RAS.^5^ The normal expression of BRAF requires dimerization, but the BRAF V600E mutation does not require dimerization and can also transmit signals; therefore, it can bypass the feedback inhibition caused by the ERK pathway.^2^, ^5^

## 3 BRAF STRUCTURE

**Figure 3:**
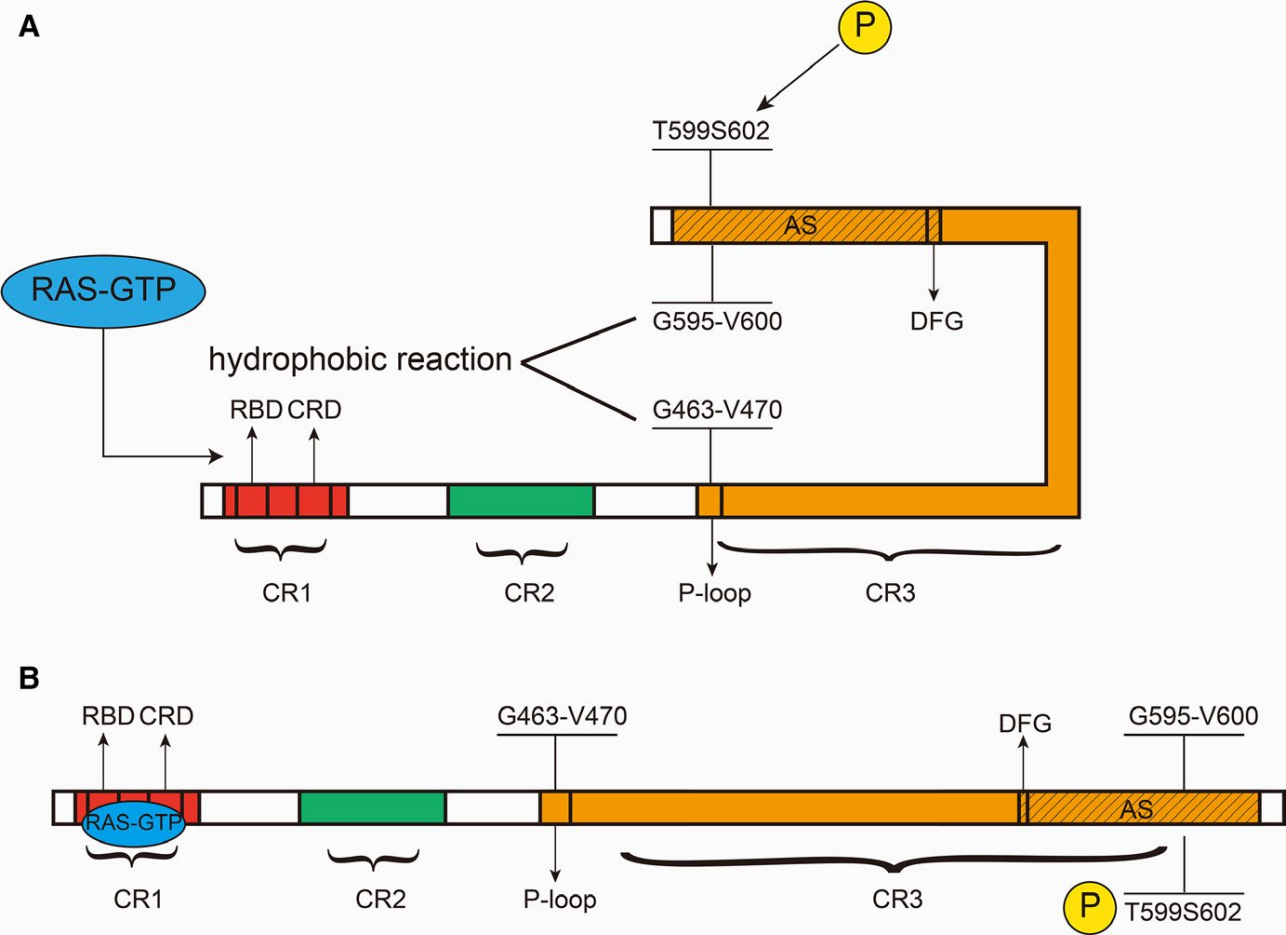
BRAF structures along with their activation mechanisms. A, BRAF consists of three parts: CR1, CR2, and CR3. CR1 includes the RBD and the CRD, both of which can combine with RAS‐GTP. CR3 includes multiple key areas. This figure lists only the P‐loop of the N‐terminal region and the AS of the C‐terminal region, where the DFG is located in the AS. When BRAF is inactive, the DFG can flip the AS, causing the AS to orient toward the P‐loop, causing a hydrophobic reaction between G595‐V600 of the AS and G463‐V470 of the P‐loop, which forms a closed conformation. B, RAS‐GTP, combined with the RBD and CRD, promotes the phosphorylation of T599 and S602. The hydrophobic reaction is disrupted, and the DFG reverses the AS, thereby activating BRAF

BRAF generally consists of two termini and three parts: the N terminus, the C terminus and CR1, CR2, and CR3. Among them, CR1 and CR2 are located at the N terminus and CR3 is located at the C terminus, which contains the kinase domain^7^, ^11^, ^12^(Figure 3A). CR1 contains two key regions, the RAS‐binding domain (RBD) and the cysteine‐rich domain (CRD), which combine with RAS‐GTP and are indispensable for cell membrane recruitment.^5^, ^13^, ^14^ An important function of CR1 is to inhibit CR3 and hence keep BRAF inactive.^13^, ^15^ When CR1 and RAS‐GTP are combined, this inhibition is relieved, and BRAF is activated (phosphorylation of the activation segment (AS) is also required).^13^ CR3 contains several key regions, namely, the P‐loop (also known as the glycine‐rich loop, located in the N‐region), an αC helix (important for the formation of BRAF‐CRAF dimers), a dimerization interface (DIF), a catalytic loop, a DFG motif, and the AS. Among them, the DFG motif is located at amino acids 594‐596, and the AS is located at amino acids 594‐623.^15^ It can be inferred that the DFG motif is located within the AS. There are two key sites on the AS, T599 and S602, and the phosphorylation of these two sites is necessary for BRAF activation.^16^ The activation of BRAF is achieved by a change in conformation. BRAF can be structurally divided into two lobes: one small lobe and one large lobe. In the BRAF inactive state, the DFG motif makes the AS orient toward the P‐loop, which causes the G595‐V600 of the AS and the G463‐V470 of the P‐loop to approach each other, forming a hydrophobic reaction, which maintains BRAF in an inactive state.^1^, ^11^, ^12^, ^17^ When combined with the RBD and CRD, RAS‐GTP stimulates the phosphorylation of T599 and S602, hence disrupting the hydrophobic reaction, which causes the DFG to flip the AS again and activate BRAF^1^, ^12^, ^16^, ^18^ (Figure 3B).

## 4 BRAF MUTATION AND ITS CLASSIFICATIONS

**Figure 4:**
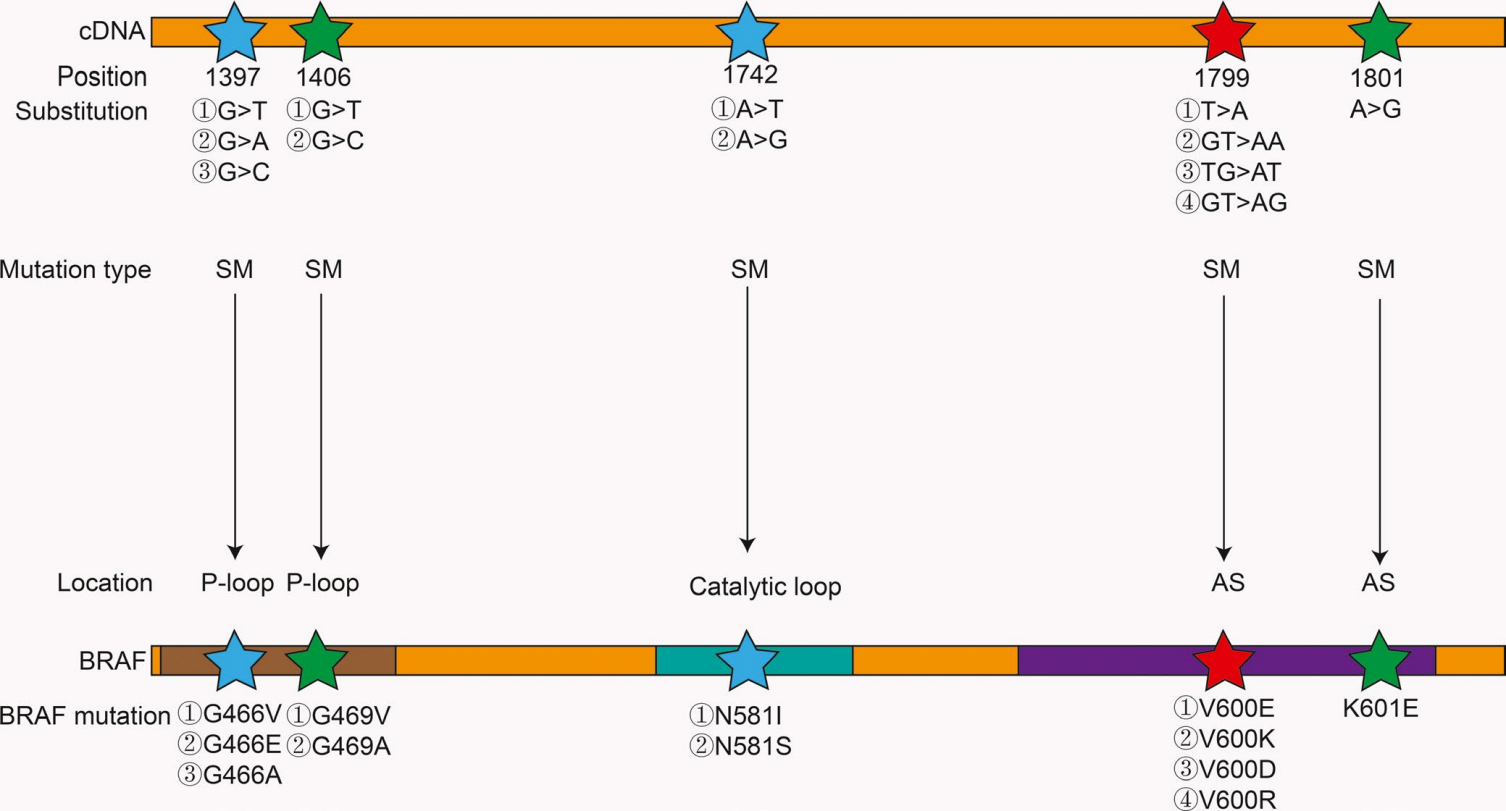
Mutation positions along with mutation results of the three BRAF mutation types. Several typical mutation positions of the three BRAF mutation types. Only the mutations in the P‐loop, catalytic loop, and AS are listed in the figure. Data source: COSMIC database. SM: substitution mutation. Red stars represent Class I mutation. Green stars represent Class II mutation. Blue stars represent Class III mutation

Most BRAF mutations occur on the P‐loop and the AS, such as the BRAF V600E mutation that occurs in the AS, and this mutation activates BRAF by destroying the hydrophobic reaction.^17^, ^19^ In contrast, some mutations may weaken BRAF activity and even make BRAF completely inactive (such as BRAFD593V). As early as 2004, BRAF mutations were classified into high, medium, and low activity mutations based on the level of BRAF mutation activity in vivo.^17^ Later, in 2017, a report further classified BRAF mutations into three types of mutations based on BRAF mutation activity, whether the mutation is dependent on RAS, whether the BRAF mutation is a monomer or a dimer, and the sensitivity to BRAF inhibitors^15^, ^20^, ^21^ (Figure 4). For patients with clinically different BRAF mutations, this classification of mutations will be of great significance for guiding treatments in the future.

Class I mutant BRAF activity is elevated and typically includes the V600E, V600K, V600D, and V600R mutations, which are RAS‐independent mutations that exist as monomers and are sensitive to BRAF inhibitors.^15^, ^20^ BRAF V600E destroys the hydrophobic reaction, thus activating BRAF. On the other hand, wild‐type BRAF activation requires dimerization, but V600E can form a salt bridge with K507. This salt bridge mimics the conformational change during dimerization, so V600E can activate MEK in a monomeric state.^15^ Moreover, BRAF V600E is not affected by the inhibitory reaction between CR1 and CR3.^13^ One typical class II BRAF mutation is K601E; BRAF activity is intermediate or elevated and is independent of RAS, similar to class I BRAF mutations. The difference between class I and class II BRAF mutations is that class II BRAF mutations exist in the form of dimers and are insensitive to BRAF inhibitors because current BRAF inhibitors, such as vemurafenib, are sensitive only to BRAF mutations in the monomeric form.^15^, ^20^ When the inhibitor combines with the first site of the dimer, the affinity for the second site is reduced by approximately 30 times. For class II mutations, the efficacy of MEK inhibitors is better than that of BRAF inhibitors, but BRAF inhibitors combined with MEK inhibitors can provide additional efficacy.^15^ Fortunately, a novel RAF inhibitor, BGB659, can inhibit BRAF dimers and monomers at the same concentration, which may bring new hope to patients with class II BRAF mutations.^20^ Class III mutations typically lead to G466V (impaired activity), D593V (completely inactive), etc, existing in the form of heterodimers, being dependent on RAS and having impaired or even kinase‐dead activity.^11^, ^17^, ^21^ Although this mutation reduces BRAF activity, it can activate MEK by activating CRAF.^17^ Unlike class I and class II mutations, RAS is active in this type of mutation because it cannot form sufficient negative feedback inhibition for RAS. Moreover, class III mutations often coexist with RAS mutations or NF1 deletions or mutations. Class III mutations are also insensitive to vemurafenib, but MEK inhibitors or ERK inhibitors, such as trametinib, may be effective. In addition, in class III mutations, RAS may also be activated by upstream RTKs, so RTK inhibitors combined with MEK inhibitors may also be effective against such mutations.^21^

**Figure 5:**
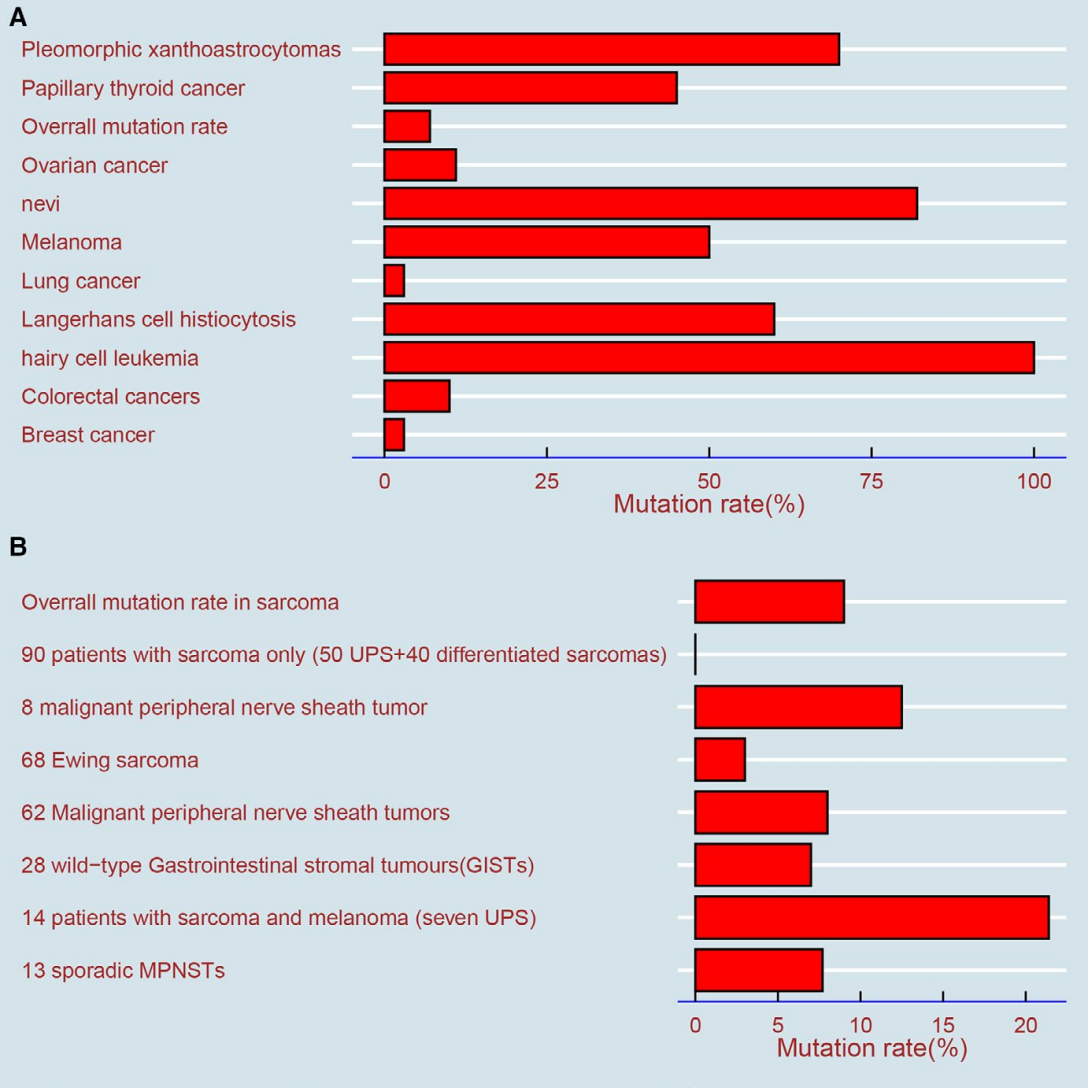
Mutation rates of BRAF in several types of sarcoma and nonsarcoma. A, The overall mutation rate of BRAF in several nonsarcomas. B, The mutation rate of BRAF in different types of sarcoma

The overall mutation rate of BRAF in malignant tumors is 7% but varies with the tumor type.^22^, ^23^ It is estimated that the BRAF mutation occurs in almost all hairy cell leukemias, at least 50% of melanomas, 40% of thyroid cancers, 10% or less of colorectal cancers, and rarely in clear cell sarcomas (CCSs) and gastrointestinal stromal tumors (GISTs)^4^, ^22^, ^24^, ^25^ (Figure 5A).

## 5 BRAF MUTATIONS IN SARCOMA

Sarcomas are rare, malignant solid tumors that originate in mesenchymal cells and can occur anywhere in the body, accounting for 1% of all malignancies in adults and 15% of malignancies in children.^26^ There are more than 50 different subtypes of sarcomas, which can be divided into soft tissue sarcomas (80%) and bone sarcomas (20%).^27^, ^28^ Gastrointestinal stromal tumors were also classified as soft tissue sarcomas in the WHO classification in 2013, although some researchers still report gastrointestinal stromal tumors and soft tissue sarcomas separately.^26^ The incidence and survival rates of sarcomas reported in different countries vary slightly. In Germany, the incidence rates of soft tissue sarcomas and bone sarcomas are 4.5 per 100 000 and 2.1 per 100 000, respectively. In addition, in terms of the age of onset, soft tissue sarcomas reach two peaks at the age of 10‐19 years and 70‐79 years, while bone sarcomas peak at only 10‐19 years. The 1‐year and 5‐year survival rates of soft tissue sarcomas are 87.8% and 66.4%, and the 1‐year and 5‐year survival rates of bone sarcomas are 91.8% and 52.9%, respectively.^29^ A report on the incidence of sarcomas in 27 EU countries showed that the overall incidence of sarcomas is 5.6 per 100 000, of which the incidence of soft tissue sarcomas is 4.7 per 100 000, and the 5‐year survival rates of soft tissue sarcomas and bone sarcomas are 58% and 62%, respectively.^30^ In China, there were approximately 39 900 new cases of soft tissue sarcoma in 2014, with an incidence rate of 2.91 per 100 000.^26^ In the United States, 13 040 people were diagnosed with soft tissue sarcoma in 2018, and approximately 5150 people died of soft tissue sarcoma.^31^ It should be noted that soft tissue sarcomas differ from visceral sarcomas slightly in the WHO classification, and histology is usually not considered in visceral sarcomas, so the total incidence of sarcomas may be underestimated.^32^

Similar to other malignant tumors, BRAF mutations also exist in sarcomas, which have been confirmed by researchers, and the overall mutation is less than 9%^24^, ^33^(Figure 5B).To our knowledge, few systematic studies on the mutation status and gene sequencing of BRAF in sarcoma have been performed; most reports have focused on case reports and treatment effects in patients treated with BRAF inhibitors.^24^ A study showed that BRAF V600E is positive in one of eight patients with malignant peripheral nerve sheath tumors (MPNSTs).^34^ In a study of genetic changes in Ewing sarcoma, the strong, positive expression of BRAF (defined as a staining score greater than or equal to 100) was only 3%, suggesting that BRAF inhibitors have limited application in Ewing sarcoma.^35^ In a study of GIST, BRAF V600E mutations were detected in two of 28 wild‐type patients.^36^ Of the 62 MPNST patients, the BRAF V600E mutations were found in five (8%) patients, which were slightly more than the 7.7% (1/13) of MPNST patients reported by another study.^37^, ^38^

The role of the BRAF mutation in sarcomas is controversial. A study analyzed 108 sarcoma samples by gene sequencing, and the data showed that the BRAF mutation was not common, suggesting that the BRAF mutation might not cause malignancy.^39^ However, another report proposed that the BRAF mutation could lead to malignancy, not just a mutation.^37^ In an analogous study, the researchers observed that 90 patients with sarcomas without a history of malignant melanoma did not have a BRAF mutation; however, BRAF mutations were present in three of 14 patients with a history of malignant melanoma, suggesting that in a poorly differentiated sarcoma with a history of melanoma, the sarcoma is likely to be melanoma and is going through the process of dedifferentiation.^24^ This conclusion is very interesting and suggests a possible relationship between melanoma and sarcoma, which may provide a new perspective for the study of BRAF mutations in sarcomas. These reports provide some references for the evaluation of BRAF mutations and gene sequencing in sarcoma to some extent, and future studies should focus on extensive, multispecimen research. Given the rarity of sarcoma and species diversity, it may take a long time to collect enough specimens, and the efforts of individual research institutions or individuals are far from enough; therefore, it may be best to combine data from research centers around the world.

## 6 SEVERAL TYPICAL BRAF INHIBITORS

Because some patients with a BRAF mutation in malignant tumors do not respond well to conventional chemotherapy, people are starting to focus on MAPK pathway target inhibition.^4^, ^40^ The application of BRAF inhibitors opens a new door for the treatment of patients with BRAF mutations (Table 1).

**Table 1:** Several BRAF inhibitors and clinical trials for sarcomas

Type	Target	Indications	Number^a^	Tumor type
Sorafenib	VEGFR, wild‐type RAF, CRAF, angiogenesis	hepatocellular carcinomarenal cell carcinoma (RCC)	NCT00880542 (terminated)	sarcoma
			NCT00864032 (completed)	Soft tissue sarcoma
			NCT00541840 (unknown)	Soft tissue sarcoma
			NCT00217620 (completed)	sarcoma
			NCT00822848 (completed)	sarcoma
			NCT00287495 (terminated)	Kaposi's sarcoma
			NCT00837148 (completed)	Sarcoma, Synovial sarcoma, leiomyosarcoma, malignant peripheral nerve sheath tumor
			NCT00406601 (completed)	sarcoma
			NCT02050919 (active, not recruiting)	sarcoma
			NCT00874874 (unknown)	sarcoma
			NCT00245102 (completed)	sarcoma
			NCT00330421 (completed)	Ewing sarcoma, osteosarcoma, soft tissue sarcoma
			NCT01005797 (completed)	renal cancer, non‐small cell lung cancer, soft tissue sarcoma
			NCT01518413 (completed)	Rhabdomyosarcoma and other soft tissue sarcoma, Ewing sarcoma, osteosarcoma
			NCT01946529 (Active, not recruiting)	Ewing sarcoma
			NCT01804374 (completed)	osteosarcoma
			NCT00889057 (completed)	osteosarcoma
vemurafenib (PLX4032) and its analog PLX4720	BRAF V600E	melanoma	NCT03220035 (Recruiting)	Ewing sarcoma, osteosarcoma, soft tissue sarcoma
			NCT03155620 (Recruiting)	Ewing sarcoma, osteosarcoma, soft tissue sarcoma
Dabrafenib (GSK2118436)	BRAF V600E, V600K and V600G	Melanoma, thyroid carcinomas	NCT03784014(Not yet recruiting)	soft tissue sarcoma
Encorafenib(LGX818)	BRAF V600E, V600D, V600K, BRAF wild type, CRAF	Melanoma	None	None

^a^ Data source: https://clinicaltrials.gov/.

Sorafenib was the first RAF inhibitor approved in clinical practice.^7^, ^23^ It was previously believed that CRAF was an important effector of RAS. Therefore, sorafenib was initially developed as a CRAF inhibitor.^6^, ^41^ Sorafenib inhibits several targets, including VEGFR, wild‐type RAF, and CRAF. Subsequent studies have shown that sorafenib mainly inhibits angiogenesis by inhibiting VEGFR, which exerts an antitumor effect.^6^, ^41^ However, because of off‐target effects, the inhibition of RAF is very limited.^6^, ^23^ Therefore, sorafenib is mainly used for renal cell carcinoma and hepatocellular carcinoma, and its effect on melanoma mainly caused by a BRAF mutation is not good.^6^, ^7^ In addition, sorafenib inhibits CRAF activity eight times as much as it inhibits BRAF.^23^ Given that BRAF accounts for 95% of all RAF mutations, it also restricts the use of sorafenib in melanoma.

Vemurafenib (PLX4032) and its analogue PLX4720 can be highly selective in inhibiting BRAF V600E.^42^ It was first synthesized in 2005 and was approved for the treatment of patients with melanoma with BRAF V600E in the United States and the European Union in August 2011 and February 2012, respectively.^41^, ^43^ The inhibitory mechanism of vemurafenib is directly related to its structure; it can directly inhibit the DFG motif, which is related to BRAF activation. At the same time, its inhibition is very characteristic. Different from MEK inhibitors, which can inhibit both tumor cells and normal cells at the same time, vemurafenib inhibits only tumor cells with the BRAF V600E mutation.^7^, ^40^, ^41^ This may actually be an advantage of vemurafenib—it does not cause an adverse reaction that inhibits the ERK pathway in normal cells.^40^ In wild‐type BRAF cells and in cells with RAS mutations treated with vemurafenib, the ERK pathway can be activated paradoxically.^2^, ^6^, ^7^, ^41^ However, this phenomenon does not appear in BRAF V600E‐mutant cells. It should be noted that this phenomenon occurs not only with vemurafenib but also with other RAF ATP competitive inhibitors.^6^ This also limits the extent of vemurafenib use, which requires the presence of BRAF mutations to be identified before the drug is administered.^7^ In addition, BRAF inhibitors are also used to treat colorectal cancer, whereas its efficacy is significantly lower than that of melanoma. Only 5% of colorectal cancer patients achieve a response, which is mainly related to reactivation of the MAPK pathway caused by EGFR.^2^, ^44^ Thus, patients with colorectal cancer may be treated with other drugs, such as EGFR inhibitors.^41^, ^44^ There have been reports about the efficacy of vemurafenib in thyroid cancer, hairy cell leukemia, and lung cancer with BRAF mutations.

Another BRAF inhibitor, dabrafenib (GSK2118436), was approved by the FDA for the treatment of melanoma on 29 May 2013.^41^ Common adverse reactions, which includes squamous cell carcinoma, are similar to those observed with vemurafenib.^7^ Dabrafenib is 80 times more potent than vemurafenib, and studies have shown that dabrafenib also has effects on V600K and V600G mutations but not on K601E.^7^

The most recent BRAF inhibitor is encorafenib. It is characterized by a longer dissociation half‐life (30 hours), stronger effects, and less paradoxical MAPK pathway activation than either vemurafenib or dabrafenib, which indicates that encorafenib has a longer inhibitory effect and fewer adverse reactions.^45^, ^46^, ^47^ On 27 June 2018, the combination of encorafenib and binimetinib (a kind of MEK inhibitor) was approved by the FDA to treat patients with unresectable or metastatic melanoma with a BRAF V600E or V600K mutation.^48^ The European Medicines Agency (EMA) also approved the combination therapy for melanoma.^49^ A clinical trial called COLUMBUS has shown that this kind of combination has the longest median progression‐free survival (PFS) of 14.9 months and a median overall survival of 33.6 months compared with other BRAF‐MEK combination therapies, with favorable adverse events.^50^, ^51^

## 7 EFFICACY OF BRAF MUTATION INHIBITORS IN SARCOMA

A number of studies have reported that melanoma patients with the BRAF V600E mutation treated with BRAF inhibitors could achieve complete or partial response according to the Response Evaluation Criteria in Solid Tumors (RECIST). However, to our knowledge, there are few clinical trials about the treatment of sarcoma patients with the BRAF V600E mutation treated with BRAF inhibitors, and most reports of sarcoma patients treated with BRAF inhibitors are case reports (Table 2). A study on nonmelanoma patients with a BRAF mutation treated with vemurafenib found that the response rates of non‐small cell lung cancer and Erdheim‐Chester disease or Langerhans cell histiocytosis with a BRAF mutation were 42% and 43%, respectively.^44^ A case report recorded a patient with a GIST. After surgical resection, imatinib (a kind of tyrosine kinase inhibitors) treatment failed, and the patient switched to dabrafenib; the tumor showed 14%, 18%, and 20% shrinkage at weeks 6, 15, and 24, respectively.^52^ In addition, the comforting effect of BRAF inhibitor was also observed in the treatment of Langerhans cell sarcoma and histiosarcoma with the BRAF V600E mutation.^53^, ^54^ A patient with an MPNST harboring the BRAF V600E mutation who achieved a modest response after treatment with sorafenib and had a significant effect after treatment with vemurafenib was also reported.^55^ Another study reported a patient with a high‐grade spindle cell sarcoma with lung metastasis. After the inefficacy of dacarbazine, docetaxel, and radiotherapy, gene sequencing revealed the presence of the BRAF V600E mutation. After treatment with vemurafenib and trametinib, both primary and lung metastases were reduced.^56^ A preclinical trial for the treatment of sarcoma with vemurafenib showed significant inhibition in the SA‐4 cell line (representing liposarcoma), as well as an inhibitory effect on the SW‐872 cell line, and found that the primary mechanism of vemurafenib is to cause G1 cell cycle arrest, and apoptosis is only a small part of the mechanism. In addition, the intermittent use of vemurafenib showed poor efficacy, indicating that the continuous use of vemurafenib is necessary for improved efficacy.^43^ In addition, other case reports have also reported that BRAF inhibitors are effective for sarcoma (Table 2). All of these studies have a common feature, that is, the BRAF mutation is present, and all of them have achieved various degrees of a response after the use of BRAF inhibitors. This finding also provides insight into the prospect of the application of BRAF inhibitors in sarcomas and raises the possibility that as long as there are BRAF mutations in sarcomas, there is the possibility of using BRAF inhibitors.

**Table 2:** Several cases reports of sarcomas treated with BRAF inhibitors

Type	Early treatment	Early treatment efficacy	Mutation type	BRAF inhibitor	Efficacy of BRAF inhibitor	Adverse events after use of BRAF inhibitor	References
Gastrointestinal Stromal Tumor	Surgical resection, adjuvant imatinib therapy, adjuvant sunitinib, MEK inhibitor.	Tumor progress	BRAF V600E	dabrafenib	The tumor showed 14%, 18%, and 20% shrinkage at week 6, 15, and 24, respectively.	grade 2 rash and acrochrodons, grade 1 fatigue and hyperkeratosis	[52]
Malignant peripheral nerve sheath tumor	Radiation therapy	Metastasize	BRAF V600E	sorafenib initially, vemurafenib thereafter	The tumor shrank slightly but increased late with sorafenib. All of the tumors shrink treatment with vemurafenib	Not mentioned with sorafenib. Rash and fever with vemurafenib treatment	[55]
high‐grade spindle cell STS with pulmonary metastases	Surgical debridement, chemotherapy, radiation therapy	tumor progress	BRAF V600E	Dabrafenib plus trametinib	dramatic response	Not mentioned	[56]
histiocytic sarcoma	chemotherapy	tumor progress	BRAF V600E	Die before use	‐	‐	[25]
histiocytic sarcoma	Steroid therapy	Not mentioned	BRAF V600E	vemurafenib	dramatic clinical improvement	asthenia, vomiting, and dysgeusia	[89
Clear cell sarcoma	Chemotherapy, surgery, radiation therapy	disease progress	BRAF V600E	Die before use	‐	‐	[90]
Clear cell sarcoma	chemotherapy	Relapse disease progress	BRAF V600E	vemurafenib	complete response	dermatological toxicity, dermatological toxicity	[91]

## 8 MECHANISMS OF RESISTANCE TO BRAF MUTATION INHIBITORS

**Figure 6:**
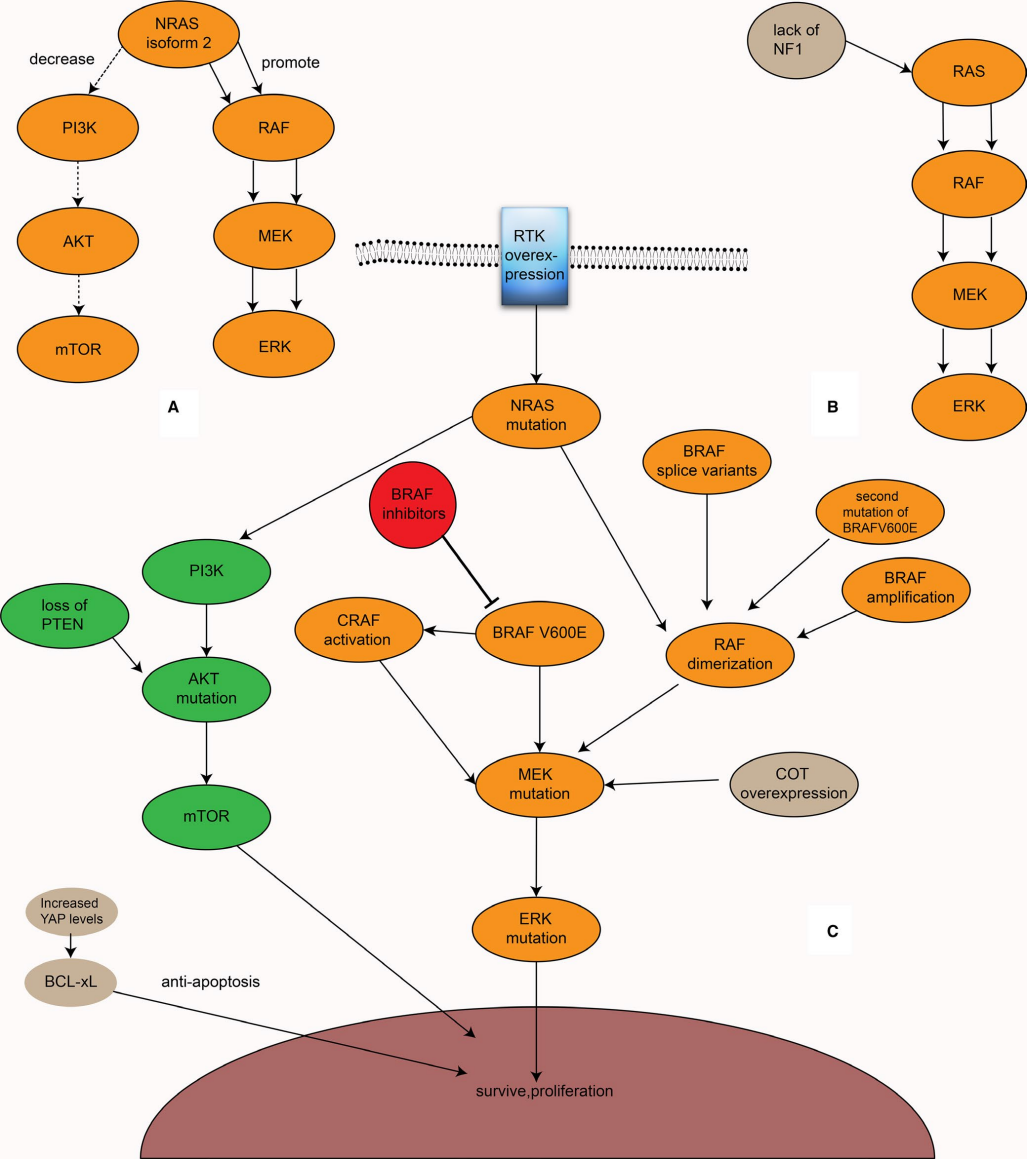
The resistance mechanism to vemurafenib. A, In general, these resistance mechanisms can be divided into two categories: MAPK‐dependent pathway paradoxical activation and PI3K‐AKT‐mTOR activation. B, NRAS isoform 2 can promote the MAPK signaling pathway and decrease the PI3K‐AKT‐mTOR signaling pathway, which confers resistance. C, NF1 regulates RAS in a manner of negative feedback, and its lack promotes the MAPK signaling pathway

BRAF inhibitors also have drug resistance, which can be divided into inherent resistance and acquired resistance.^2^, ^41^, ^57^ Most patients develop resistance within 1 year after treatment with vemurafenib initially.^9^, ^58^ For some people, BRAF inhibitors do not work from the initial treatment.^59^, ^60^ Thus far, more than ten resistance mechanisms have been described, and there could be multiple resistance mechanisms in the same patient^61^, ^62^, ^63^ (Figure 6A‐C). In general, these resistance mechanisms can be divided into two categories: the MAPK‐dependent pathway paradoxical activation and the activation of alternative pathways, mainly PI3K‐AKT‐mTOR (Figure 6C). Among them, the former accounts for the majority of drug resistance. Despite these findings, the resistance mechanism to BRAF inhibitors remains unclear in a considerable number of patients.^42^

### 8.1 MAPK‐dependent pathway paradoxical activation

A report found a second BRAF V600E mutation, BRAF V600E L514V, in a patient with a BRAF V600E‐mutant left temporal lobe brain tumor who relapsed after treatment with dabrafenib. Moreover, this mutation was not detected before recurrence, suggesting that BRAF inhibitor resistance can be generated by a second mutation in BRAF. This mutation can increase RAF dimer formation and promote ERK pathway signal expression.^64^ In the BRAF gene, a kind of splice variant that deletes a part of the exon that removes the RBD participates in the resistance to BRAF inhibitors by increasing the tendency to form homologous dimers and enhancing the MAPK signaling pathway.^65^, ^66^, ^67^ A study supported that the AGAP3‐BRAF fusion protein, which lacks the RBD, may be a novel resistance mechanism.^61^ Some researchers also demonstrated that BRAF, when inhibited, can be transformed into CRAF or ARAF through a kinase transformation that continues to activate MAPK, leading to resistance to BRAF inhibitors.^68^ In addition, in RAS mutations, BRAF can bind to CRAF when BRAF is inhibited, thereby promoting the MAPK pathway. However, it should be noted that in this case, BRAF does not directly activate MEK but activates CRAF to activate MEK.^19^ This can lead to the activation of MAPK by BRAF inhibitors paradoxically. However, this activation mechanism does not occur when treated with RAF paninhibitors, such as sorafenib, which inhibits BRAF as well as CRAF. NRAS upregulation, which can enhance the dimerization of RAF and contribute to resistance, is also relevant.^2^, ^69^ In addition to the BRAF V600E second mutation, some splice variants, and NRAS upregulation, BRAF amplification also likely plays a role in the resistance mechanism by the formation of dimers.^2^, ^59^ Another study showed that NRAS isoform 2 (a kind of NRAS isoform) participates in resistance to BRAF inhibitors by promoting the MAPK signaling pathway and decreasing the PI3K‐AKT‐mTOR signaling pathway, which suggests a potential connection between the MAPK and the PI3K signaling pathways^70^ (Figure 6A). In addition, the overexpression of cancer Osaka thyroid (COT), which can promote MEK and ERK activation in a BRAF‐independent manner, is also related.^2^, ^66^, ^71^ The lack of NF1, which regulates RAS in a manner of negative feedback (Figure 6B), MEK1/2 mutation, and ERK mutation also confer resistance to BRAF inhibitors.^59^, ^66^, ^70^, ^72^ Although MAPK reactivation is a significant mechanism of BRAF inhibitor resistance, resistant tumors are still sensitive to ERK inhibitors.^63^, ^73^ Moreover, not all patients who are resistant to BRAF inhibitors have MAPK activation, and some patients still show MAPK inhibition; therefore, the PFS rate of the former is higher than that of the latter.^74^

### 8.2 PI3K‐AKT‐MTOR activation

The PI3K‐AKT‐mTOR signaling pathway is also activated by RAS and participates in various physiological processes, such as cell proliferation and differentiation. In this signaling pathway, the lack of PTEN, which inhibits AKT, or a mutation in AKT can cause resistance to BRAF inhibitors^63^, ^66^, ^75^ (Figure 6C). In contrast, the activation or overexpression of an RTK, which usually activates both the MAPK and PI3K‐AKT‐mTOR signaling pathways, including EGFR, MET, PDGFRβ, and IGF1R, can exist in BRAF inhibitor‐resistant melanoma cells by activating PI3K‐AKT‐mTOR.^42^, ^59^, ^69^, ^74^, ^76^ Among them, EGFR overexpression is an important mechanism of resistance to BRAF inhibitors in colorectal cancer patients.^2^

### 8.3 Other mechanisms

In addition, a change in the tumor microenvironment also plays an important role in the resistance mechanism.^42^, ^74^, ^77^ For example, the presence of macrophages may be associated with the development of BRAF inhibitor resistance.^75^ In addition, hepatocyte growth factor (HGF), a regulator of BAD secreted by mesenchymal cells, can prevent BAD dephosphorylation and thus participate in BRAF inhibitor resistance via the PI3K‐AKT‐mTOR signaling pathway.^57^, ^78^ HGF and its receptor MET also confer resistance to BRAF inhibitors.^77^ In several types of tumor cells, YAP can increase the expression of BCL‐xl, which is antiapoptotic, leading to RAF inhibitor resistance. Furthermore, the increased expression of YAP can lead to resistance to MEK inhibitors. The inhibition of both YAP and MAPK signaling pathways can achieve improved efficacy.^60^

## 9 COMBINATION WITH OTHER TARGETED THERAPIES

Because BRAF inhibitor monotherapy is vulnerable to drug resistance, attention has focused on the combination of BRAF inhibitors with other drugs, including other gene inhibitors of the MAPK and PI3K‐AKT‐mTOR signaling pathways, such as EGFR inhibitors, PI3K inhibitors, mTOR inhibitors, MEK inhibitors, RTK inhibitors, HGF inhibitors, and MET inhibitors.^57^ Currently, three BRAF inhibitors have been approved by the FDA for combination use with MEK inhibitors in the treatment of melanoma—vemurafenib plus cobimetinib, dabrafenib plus trametinib, and encorafenib plus binimetinib; among them, dabrafenib plus trametinib was the first combination approved by the FDA.^75^, ^79^, ^80^

In the coBRIM clinical trial, the median PFS (mPFS) of patients treated with vemurafenib plus cobimetinib and vemurafenib plus placebo was 12.3 months and 7.2 months, respectively, the median overall survival (mOS) was 22.3 months and 17.4 months, respectively, and the overall response rate (ORR) was 70% (complete response (CR) 16%+ partial response (PR) 54%) and 50% (CR 10%+PR 40%), respectively (Table 3). The rates of cutaneous squamous adenocarcinoma and keratoacanthoma were 6% and 20%, respectively.^81^ From these data, it can be seen that regardless of whether mPFS, OS, or ORR is considered, for vemurafenib, its combination with a MEK inhibitor regimen is more effective than its monotherapy regimen, and the incidence of severe adverse reactions of skin secondary tumors in the combined group is significantly lower than that in the monotherapy group. In the clinical trials COMBI‐v and COMBI‐d, the combination of dabrafenib and trametinib was also more effective than that of vemurafenib or dabrafenib monotherapy, as seen in the coBRIM trial^82^, ^83^, ^84^, ^85^ (Table 3).

**Table 3:** The results of coBRIM, COMBI‐v, COMBI‐d, and COLUMBUS part one clinical trials

The results of coBRIM clinical trial^81^		
	Vemurafenib plus cobimetinib	Vemurafenib plus placebo
mPFS(mo)	12.3	7.2
mOS(mo)	22.3	17.4
ORR	70%((CR16%+PR54%)	50%(CR10%+PR40%)
Incidence of cutaneous squamous adenocarcinoma and keratoacanthoma	6%	20%

The results of COMBI‐v clinical trial^86^		
	Dabrafenib plus trametinib	Vemurafenib monotherapy
mPFS(mo)	11.4	7.3
mOS(mo)	25.6	18
ORR	64%(CR13%+PR51%)	51%（CR8%+PR44%）
Incidence of cutaneous squamous adenocarcinoma and keratoacanthoma	1%	18%

The results of COMBI‐d clinical trial^86^		
	Dabrafenib plus trametinib	Dabrafenib plus placebo
mPFS(mo)	11	8.8
mOS(mo)	25,1	18.7
ORR	67%(CR10%+PR56%)	51%(CR9%+PR43%)
Incidence of cutaneous squamous adenocarcinoma and keratoacanthoma	2%	9%

The part one results of COLUMBUS clinical trial^50^, ^87^			
	Encorafenib plus binimetinib	Encorafenib	Vemurafenib
mPFS(mo)	14.9	9.6	7.3
mOS(mo)	33.6	23.5	16.9
ORR	63%(CR11%+PR52%)	51%(CR7%+PR44%)	40%(CR8%+PR32%)
squamous cell cancers	3%	8%	17%

In June 2019, the latest follow‐up data on the COMBI‐v and COMBI‐d clinical trials were reported.^86^ The follow‐up data of the dabrafenib plus trametinib groups in the two clinical trials were combined (a total of 563 patients, including 352 in COMBI‐v and 211 in COMBI‐d), with an mPFS of 11.1 months and a mOS of 25.9 months. The PFS rate of 3, 4, and 5 years was 23%, 21%, and 19%, respectively, and the OS rate of 3, 4, and 5 years was 44%, 37%, and 34%, respectively. Moreover, the survival curve between 3 and 5 years began flatten, suggesting that the patient's condition began to stabilize, and further follow‐up became meaningful. Lactate dehydrogenase (LDH) is an important prognostic factor, and the prognosis of subgroups with different LDH levels is significantly different. Specifically, the 5‐year PFS rates for patients with an LDH level below the normal upper limit and above the normal upper limit were 25% and 8%, respectively, and the 5‐year OS rates were 43% and 16%, respectively. Furthermore, patients with LDH levels within the normal range and patients with less than three metastases were considered; the 5‐year PFS and 5‐year OS rates were 31% and 55%, respectively, which showed a significant improvement compared with the 5‐year PFS rate of 19% and the 5‐year OS rate of 34% in the whole population. The 5‐year PFS and 5‐year OS rates were particularly encouraging among patients who achieved a CR, at 49% and 71%, respectively. These data indicate that CR is a good prognosis for patients and that LDH indicates a poor prognosis. Additionally, in COMBI‐v, the 5‐year PFS and 5‐year OS rates of the vemurafenib monotherapy group were 9% and 23%, respectively; in COMBI‐v, the 5‐year PFS and 5‐year OS rates in the dabrafenib plus placebo group were 13% and 27%, respectively.

In the first part of the COLUMBUS clinical trial (the purpose of the second part was to determine the role of binimetinib in combination therapy by comparing the encorafenib plus binimetinib group with the encorafenib monotherapy group; the results have not yet been reported), the mPFS of the encorafenib plus binimetinib combined treatment group, the encorafenib monotherapy group, and the vemurafenib monotherapy group were 14.9 months, 9.6 months, and 7.3 months, respectively, and the OS was 33.6 months, 23.5 months, and 16.9 months, respectively. The ORR was 63% (CR 11%+PR 52%), 51% (CR 7%+PR 44%), and 40% (CR 8%+PR 32%), respectively.^50^, ^87^ It can be seen that the encorafenib plus binimetinib group experienced the longest mPFS and OS among the three combination treatment groups of encorafenib plus binimetinib, vemurafenib plus cobimetinib, and dabrafenib plus trametinib. Therefore, encorafenib plus binimetinib has good application prospects. However, clinical trials that directly compare the efficacy of the three treatment regimens are also required.

In addition, regarding the efficacy of vemurafenib, the data from the three clinical trials (COLUMBUS, COMBI‐v, and coBRIM) are not very different; the mPFS of the three groups was 7.3, 7.3, and 7.2 months, respectively, and the mOS was 16.9, 18, and 17.4 months, respectively.

It is not difficult to see from the results of these clinical trials that in malignant melanoma, the combination of BRAF inhibitors and MEK inhibitors cannot only improve OS and PFS but also delay the recurrence and reduce the incidence of drug‐related adverse reactions, especially the incidence of secondary skin tumors. Therefore, it is worth determining whether the combination of BRAF inhibitors and MEK inhibitors can have the same effect in sarcomas.

Notably, MEK inhibitor monotherapy is not effective in patients who have already developed resistance to BRAF inhibitors, possibly because of the cross‐resistance between BRAF inhibitors and MEK inhibitors.^80^ In addition, resistance still occurs when both BRAF inhibitors and MEK inhibitors are combined. Most patients develop resistance within 3 years after receiving the combination of vemurafenib plus cobimetinib and dabrafenib plus trametinib, and some resistance mechanisms are the same as those of BRAF monotherapy, such as MEK mutation and BRAF amplification.^46^, ^62^ Therefore, future studies should focus on how to overcome resistance in the setting of their combination. The use of vemurafenib combined with EGFR inhibitors has been shown to be effective in preclinical models.^88^ The combination of BRAF inhibitors and PI3K‐AKT‐mTOR inhibitors exhibits an excellent response.^75^ A study showed that simultaneous treatment with BRAF, EGFR, and MEK inhibitors can achieve satisfactory outcomes in colorectal cancer patients with BRAF V600E.^79^ In addition, heat shock protein 90 (HSP90) inhibitors (XL888) have been shown to overcome BRAF inhibitor resistance, and their combined application with BRAF inhibitors is currently in clinical trials.^80^

## 10 CONCLUSION

In general, *BRAF* is a key gene involved in tumorigenesis, and its activation is closely related to conformational changes. The BRAF mutation frequency varies in different tumors, and the three mutation types have different properties and characteristics, which have important guiding significance for the clinical use of BRAF inhibitors. Given the susceptibility of monotherapy to resistance, there have been exciting results obtained from combining BRAF inhibitors with other targeted inhibitors, such as MEK inhibitors. In sarcomas, the study of BRAF has not been sufficient. Current reports mainly focus on the efficacy of single patients using BRAF inhibitors and lack large‐scale clinical trials. Future clinical trials on sarcomas could greatly benefit patients with sarcoma.

## CONFLICT OF INTEREST

The authors declare that they have no conflict of interest.

## AUTHORS' CONTRIBUTIONS

HTL drafted the manuscript. NN, SH, XYL, and JLY revised the manuscript. All the authors approved the final manuscript.

## Data Availability

Data sharing is not applicable to this article as no new data were created or analyzed in this study.
